# Role of maspin in cancer

**DOI:** 10.1186/2001-1326-2-8

**Published:** 2013-03-07

**Authors:** Rossana Berardi, Francesca Morgese, Azzurra Onofri, Paola Mazzanti, Mirco Pistelli, Zelmira Ballatore, Agnese Savini, Mariagrazia De Lisa, Miriam Caramanti, Silvia Rinaldi, Silvia Pagliaretta, Matteo Santoni, Chiara Pierantoni, Stefano Cascinu

**Affiliations:** 1 Medical Oncology Unit, Università Politecnica delle Marche, Azienda Ospedaliero-Universitaria Ospedali Riuniti Umberto I, GM Lancisi, G Salesi di Ancona, Via Conca, Ancona, 71-60126, Italy

**Keywords:** Maspin, Serine protease, Prognosis

## Abstract

Maspin (mammary serine protease inhibitor), is a member of the serine protease inhibitor/non-inhibitor superfamily. Its expression is down-regulated in breast, prostate, gastric and melanoma cancers but over-expressed in pancreatic, gallbladder, colorectal, and thyroid cancers suggesting that maspin may play different activities in different cell types. However, maspin expression seems to be correlated with better prognosis in prostate, bladder, lung, gastric, colorectal, head and neck, thyroid and melanoma cancer. In breast and ovarian cancer maspin significance is associated with its subcellular localization: nucleus maspin expression correlates with a good prognosis, whilst in pancreatic cancer it predicts a poor prognosis. Since tumor metastasis requires the detachment and invasion of tumor cells through the basement membrane and stroma, a selectively increased adhesion by the presence of maspin may contribute to the inhibition of tumor metastasis. Furthermore the different position of maspin inside the cell or its epigenetic modifications may explain the different behavior of the expression of maspin between tumors. The expression of maspin might be useful as a prognostic and possibly predictive factor for patients with particular types of cancer and data can guide physicians in selecting therapy. Its expression in circulating tumor cells especially in breast cancer, could be also useful in clinical practice along with other factors, such as age, comorbidities, blood examinations in order to select the best therapy to be carried out. Focusing on the malignancies in which maspin showed a positive prognostic value, therapeutic approaches studied so far aimed to re-activate a dormant tumor suppressor gene by designed transcription factors, to hit the system that inhibits the expression of maspin, to identify natural substances that can determine the activation and the expression of maspin or possible “molecules binds” to introduce maspin in cancer cell and gene therapy capable of up-regulating the maspin in an attempt to reduce primarily the risk of metastasis.

Further studies in these directions are necessary to better define the therapeutic implication of maspin.

## Review

### Introduction

Maspin (mammary serine protease inhibitor), is a member of the serine protease inhibitor/non-inhibitor superfamily (serpin), like plasminogen activator inhibitors 1 and 2 and α1-antitrypsin. Maspin gene is located on chromosome 18q21.3–q23 and it was identified for the first time in 1994.

Maspin expression is down-regulated in breast, prostate, gastric and melanoma cancers but over-expressed in pancreatic, gallbladder, colorectal, and thyroid cancers suggesting that maspin may play different activity in different cell types. These conflicting observations might be explained by distinct subcellular localization of maspin in cancer cells (cytoplasmic, nuclear or both cytoplasmic-nuclear expression); by interactions with extracellular matrix and its structure and epigenetic modifications [1-4].

A characteristic feature of serpin structure, is a reactive center loop (RCL), a peptide stretch that is located 9–15 residues amino-terminal to the reactive site peptide bond. RCL allows the reactive site to present an optimal configuration for binding, and subsequent inhibiting target protease.

**Figure 1:**
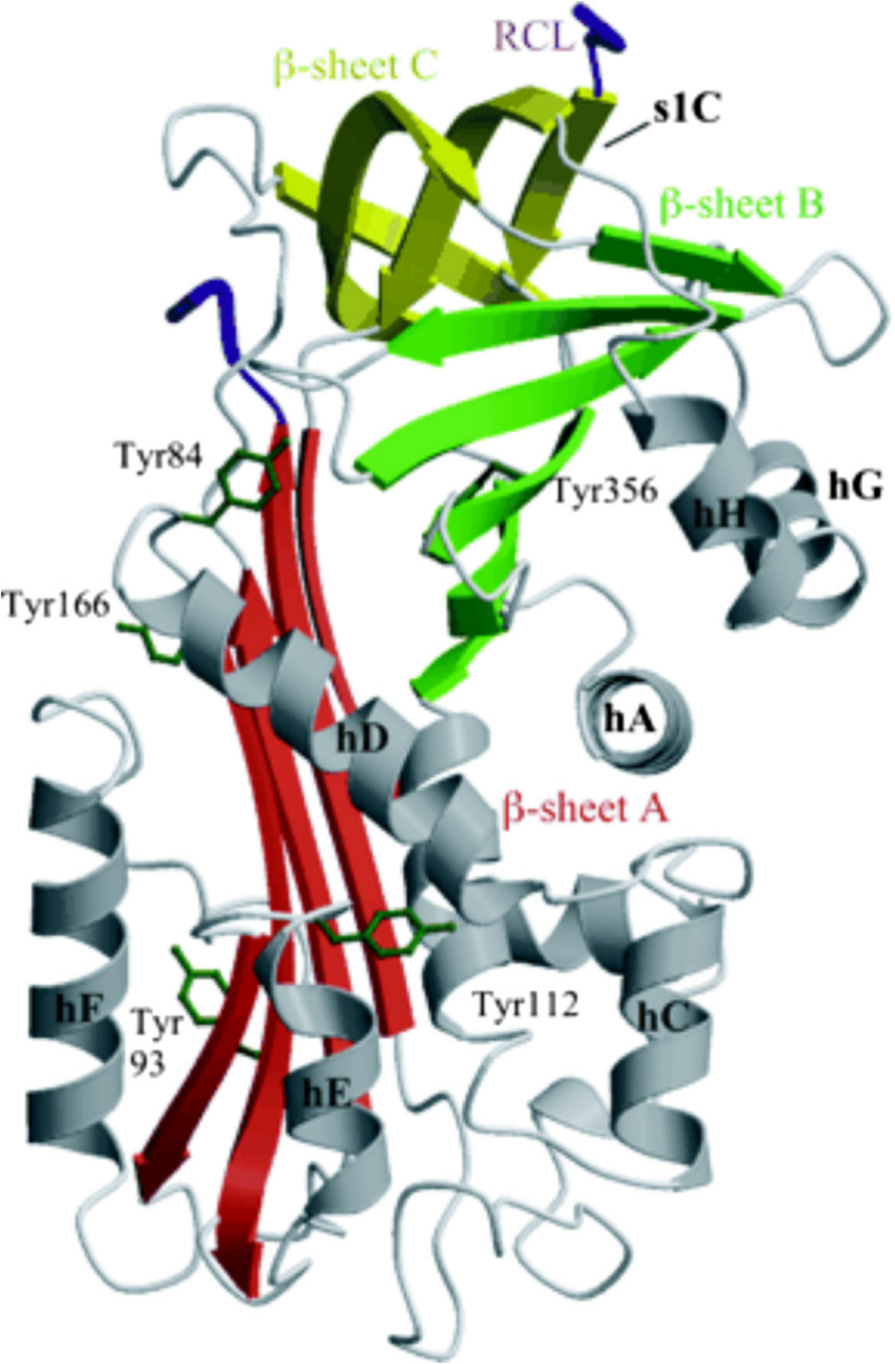
The x-ray crystal structure of maspin.

The conformational change is known as the “stressed-to-relaxed” transition. Maspin, however, contains a relatively short, divergent, not highly conserved, hydrophobic RCL not capable of undergoing this transition. Collectively, these properties place maspin into the non-inhibitorycategory of the large serpin superfamily and shift the focus away from attempting to identify a target protease as explanation for the biological activities of maspin (Figure 1) [5-7].

Another interesting aspect of Maspin pertains to the G α-helix (G-helix), an internal salt bridge or the P1 position of the reactive center loop. The Maspin G-helix is capable of an “open and closed” conformational change inducing redistribution of charged residues within the molecule.

An intact G-helix is absolutely required for the effect of maspin on cell migration, and the effect of the maspin protein can be mimicked by a short peptide corresponding to this structural element. Maspin and the G-helix in isolation are reliant on α1 integrins for their effects on cell migration [8].

**Figure 2:**
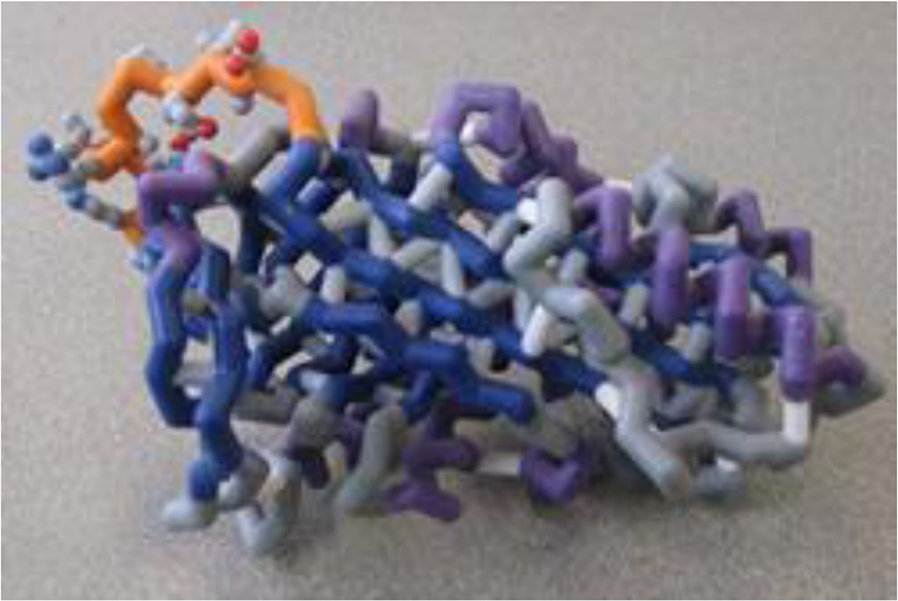
Maspin three-dimensional structure.

Furthermore the action of maspin on cell migration needed a 15-mer G-helix peptide right direct binding to the β1 integrin subunit resulting in the inactivation of β1 integrins (Figure 2) [9-11].

In fact several studies showed that Maspin elicits changes in the expression of proteins associated with the actin cytoskeleton that predict a less motile and invasive phenotype and reduced metastatic spread. Moreover, RCL appears to mediate binding to a cell surface receptor that promotes cell adhesion to type I collagen and fibronectin [12,13].

To confirm this maspin action time-lapse video microscopic studies showed that recombinant maspin dramatically also inhibited the lamellopodia extension and vectorial translation [14].

**Figure 3:**
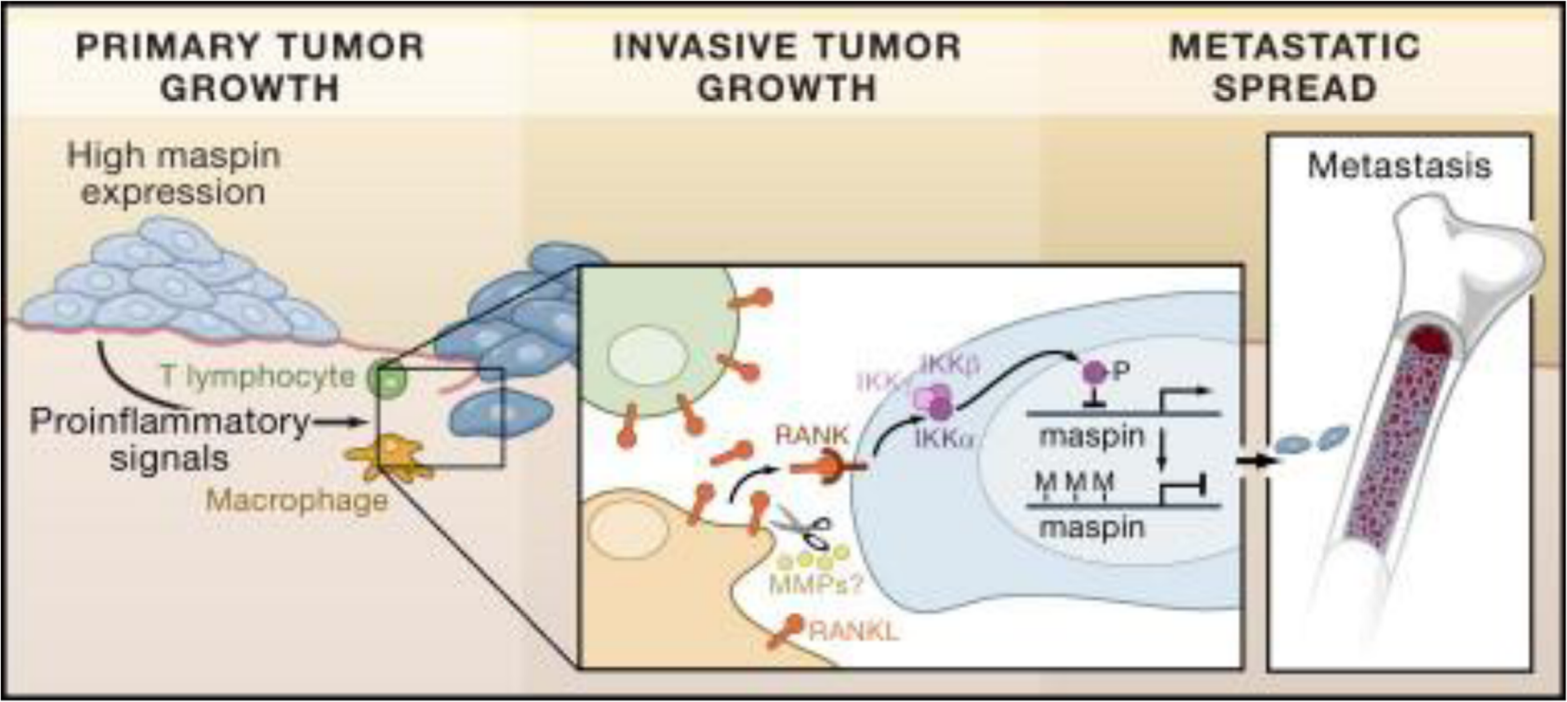
Mechanism of maspin function in most of human cancer.

Since tumor metastasis requires the detachment and invasion of tumor cells through the basement membrane and stroma, selectively increased adhesion by the presence of maspin may contribute to the inhibition of tumor metastasis (Figure 3) [15-17].

During the process of metastasis, there are consistent changes in gene expression. Studies of genes that are reduced or silenced have yielded surprising insights into in vivo mechanisms of regulating tumor metastasis. This review describes a tumor suppressor gene, Maspin, which is often silenced in cancer cells and exhibits suppressing activity against tumor growth and metastasis. Maspin has been shown to be involved in processes that are important to both tumor growth and metastasis such as cell invasion, angiogenesis, and more recently apoptosis. Hence, many efforts have been devoted to deciphering the molecular mechanism of maspin. While some insights have come from the protease inhibitory effect of maspin, more perceptive results on how maspin may function in suppressing tumor metastasis have come from studies of gene manipulation, protein interactions and global protein profiling [18].

Recent evidence indicates, however, that nuclear localization of maspin in cancer cells is necessary for its tumor suppressor activity and nuclear-localized maspin binds to chromatin are required to effectively prevent cells from metastasizing [19].

About subcellular distribution, Maspin is predominantly cytoplasmic but it also localizes to other cellular compartments and is secreted. Secreted Maspin could bind to extracellular matrix components. Alternatively, it is possible that maspin exerts its role only in the nucleus at the level of gene or chromatin regulation and thus indirectly affects the cell-matrix interaction or differentiation state and is released only as a consequence of cell damage or necrosis [20-27].

It has recently established that maspin is epigenetically regulated in tissue-specific way. Epigenetic changes of maspin expression involve cytosine methylation, histone, deacetylation, and chromatin accessibility causing loss of gene function [28-30].

Several studies showed that over-expression of maspin in gastric, pancreatic, and ovarian cancers results from promoter CpG demethylation. This clearly indicates that both methylation and demethylation of maspin promoter could regulate maspin gene expression and could guide the interpretation of overexpression/down regulation associated with negative prognosis. The promoter methylation of the maspin gene leads to gene silencing in several tumors such as breast, thyroid, skin, and colon cancer and it has been recognized as one of the most frequent mechanisms causing loss of gene function. It is noteworthy that in somatic tissues, the majority of CpG islands are methylated, and tumor cells have global DNA hypomethylation compared with their normal counterparts. Hypomethylation is involved in the progression from the premalignant to a fully developed malignancy and leads to activation of genes important for cancer development [31].

### Breast cancer

Maspin is the only pro-apoptotic serpin implicated in apoptosis regulation in breast cancer. The intracellular maspin can translocate to the mitochondria to induce cytochrome *c* release and caspase activation or modulate expression of Bcl-2 family members [32-40].

In order to confirm maspin tumor suppressor function, several authors investigated in vitro maspin expression in different tissue, from normal gland to metastatic disease. Maspin expression appears to be reduced in advanced stages of breast cancer. In fact, a significant stepwise decrease in maspin expression (and in vascular endothelial growth factor (VEGF) expression) occurred in the sequence DCIS (Ductal Carcinoma In Situ)- invasive cancer – brain-lymph-node-bone metastasis experiments. Myoepithelium, normal breast and fibrocystic change showed a strong maspin expression [41-43].

Even if Maspin absence emerges as an indicator of tumor progression and metastatic potential, recent studies showed that maspin expression was correlated with an aggressive phenotype in the breast cancer and with poor prognosis. Three hypothesis were thought for the aberrant expression of maspin in breast cancer cells: maspin gene alteration with loss of activity; a high intracellular density of maspin resulting in auto-inhibition of its function; myoepithelial cell differentiation in cancer cells could contribute to more aggressive phenotype. It is also important to investigate the different subcellular maspin expression. In fact nuclear staining was demonstrated to be significantly associated with better a prognosis than cytoplasmic staining [44,45].

Umekita et al. and Kim et al. examined, 92 and 192 invasive ductal carcinomas respectively. They reported that maspin expression was frequently observed in invasive ductal carcinoma with an aggressive phenotype (i.e. high histological grade), and it was a strong indicator of a poor prognosis [46,47].

Conversely, in order to define the significance of subcellular maspin location, Mohsin et al. performed a preliminary study assessing the associations of maspin with other established prognostic factors in invasive breast cancer. In a series of 1068 breast cancer maspin nuclear staining was significantly associated with good prognostic factors rather than cytoplasmic staining [48].

Furthermore the prognostic role of maspin was investigated by Umekita et al. and Lee et al. They examined maspin and p53 expression in 168 and 80 patients with invasive ductal carcinoma, respectively. Large tumor size, high histologic grade, positive p53 status, negative estrogen receptor or progesterone receptor status and poor prognosis were correlated with maspin expression [49-51].

Stark et al. and Maas et al., instead, investigated the potential correlations between maspin expression in primary tumor and in the metastatic sites. They showed that maspin expression is reduced in primary tumors and again decreased in metastasis [52,53].

This finding adds maspin to the list of metastasis suppressor genes potentially involved in the spread of breast cancer metastases (Table 1).

**Table 1:** Maspin expression in breast cancer

**Authors**	**Year**	**N. patients**	**Maspin expression**	**Clinical pathological features/prognosis**
Umekita et al.	2003	92 (invasive breast cancer)	18.5% positive maspin	Positive maspin in invasive breast cancer and DCIS = large tumor size, presence of comedo-necrosis and high grade
		145 (Ductal Carcinoma In Situ)	9.6%positive maspin	
		27 (atypical hyperplasia)	3.7% positive maspin	
		94 (usual hyperplasia)	0%positive maspin	
Kim et al.	2003	192 (stage I-II breast cancer)	34.4% positive maspin	Positive maspin in all type of invasive breast cancer = high grade
			36.4% positive maspin in invasive ductal carcinoma	
			7.1% positive maspin in invasive lobular carcinoma)	
Mohsin at al.	2003	1068 (breast cancer)	35% positive cytoplasm	Positive cytoplasmic maspin = negative ER and PgR, high Mib, aneuploidy
			96% positive nucleus	Positive nuclear maspin = positive ER and PgR
Umekita et al.	2002	168 (breast cancer)	27.4% positive maspin	Positive maspin = 17.4% Large tumor size, 43.4% high grade, 65.2% negative ER and PgR, 43.4% positive p53 shorter PFS and OS
			72.6% negative maspin	Negative maspin = 9% large tumor size, 19.6% high grade 35.2% negative ER and PgR 20,5% positive p53, better PFS and OS
Umekita et al.	2011	135 (triple negative breast cancer)	85.9% positive maspin	Positive maspin = 43.1% age ≤ 50 years, 80.2% high grade
			14.1% negative maspin	Negative maspin = 5.2% age ≤ 50 years, 42.1% high grade
Lee et al.	2006	80 (breast cancer)	31.3% positive maspin	Positive maspin = 24% large tumor size, 52% high grade, 80% negative PgR, Short PFS and OS
			68.7% negative maspin	Negative maspin = 7.2% large tumor size, 21.8% high grade, 41.8% negative PgR, better PFS and OS
Stark et al.	2012	16 (metastatic breast cancer)	30% positive maspin in primary tumor	
			13% positive maspin in brain metastasis	
Maass et al.	2001	45 (breast cancer)	64% positive maspin	17.7% of patients develop metastasis = 75% negative maspin
			36% negative maspin	

Nevertheless, further investigations are needed to clarify the real mechanism of the aberrant expression of maspin in breast cancer, also in order to use it as a prognostic marker in clinical practice.

### Prostate cancer

In human prostate cancer, maspin expression consistently appears to be down-regulated at the critical transition from non-invasive, low grade to high grade prostate cancer. In particular, the loss of basolateral maspin expression in benign secretory cells, the dramatically up-regulation in High Grade Prostatic Intraepithelial Neoplasia and the progressively decrease in invasive cancer are associated with maspin capability to reduce tumor growth, osteolysis and angiogenesis. Furthermore, there is an evidence that maspin inhibits prostate cancer-induced bone matrix remodeling and induces prostate cancer glandular redifferentiation [54-61].

Lovric et al. examined 34 biopsies of prostatic carcinoma. Maspin resulted expressed in the cytoplasm of basal cells of normal prostatic glands, whilst normal luminal cells were inconsistently weakly positive and it was aberrant over-expressed in prostate cancer with a predominance of nuclear presence [62].

On the other hand, Riddick et al. and Machtens et al. analyzed gene expression and clinico-pathologic features in 44 and 84 patients with prostate cancer, respectively. Maspin expression was inversely correlated with the Gleason score and positively correlated with lower tumor stage, more differentiated grade and a lower p53 protein mutation [63,64].

Zou et al. investigated Maspin expression in 97 prostate tumor specimens showing that tumor cells that exhibit histological response to neoadjuvant “hormonal treatment” showed Maspin expression. These data suggest that the androgen withdrawal may unmask Maspin expression in prostate cancer, which frequently lacks Maspin expression (Table 2) [65].

**Table 2:** Maspin expression in prostate cancer

**Authors**	**Years**	**N. patients**	**Maspin expression**	**Clinical features/prognosis**
Lovric et al.	2010	34 prostate cancer	79% positive maspin with nuclear expression	
Riddick et al.	2005	44 prostate cancer	Not found	Positive maspin = lower Gleason score
Machtens et al.	2001	84 prostate cancer	58% positive maspin	Positive maspin = OS: 78 months, PFS: 41 months, GIII-IV: 23%, N1/N2: 9% OS = 62 months, PFS = 26 months, GIII-IV = 48%, N1/N2 = 18%
			18% negative maspin	
Zou et al.	2002	97 prostate cancer	40.2% positive maspin	Positive maspin = 91.7% partial response with neoadjuvant therapy
			49.8% negative maspin	

In conclusion, Maspin expression correlates with a better prognosis and may serve as a biomarker for prostate cancer cells responding to the androgen ablation therapy.

### Bladder cancer

Recent evidences seem to correlate a low maspin expression in bladder tumors to an increased tumor cell growth both in vivo and in vitro. In particular, maspin expression was preserved in superficial bladder cancers but significantly decreased in invasive carcinomas. Within the group of invasive cancers, the authors found that maspin expression was associated with good prognosis.

Recently, Lockett et al. showed the essential role of maspin in epithelial homeostasis and that a sub-cellular location of maspin seems to reflect a distinct tumor progression pathway. In particular, the nuclear location of maspin seems to be associated with lower histological grade and longer recurrence free disease [65-79].

Acikalin et al. evaluated the clinical significance of maspin in patients with T1 bladder cancer. They showed that maspin expression was associated with a longer time to recurrence and progression-free survival (PFS) than maspin-negative group [80].

Furthermore Kramer et al. investigated the role of maspin in transitional cell carcinoma of the bladder as well as its prognostic impact. Specimens from 162 non-muscle invasive bladder cancer patients treated by transurethral resection were examined. They showed that a low maspin protein expression was correlated with a higher incidence of tumor progression and emphasized a possible clinical role of this novel tumor suppressor gene in transitional cell carcinoma of the bladder [81].

In another study, Friedrich et al. analyzed the expression patterns of maspin in 110 pTa/pT1 urothelial carcinoma of the bladder and compared them with microvessel density (MVD) evaluated by CD105 and CD34. They found a decreased Maspin expression in a large portion of pTa/pT1 bladder tumors and shorter PFS with an increase of MVD in maspin negative cancers [82].

Again, Sugimoto et al. evaluated maspin expression in 65 series of bladder cancer. Maspin expression was significantly correlated with the development of muscle invasive bladder cancer. Their contradictory results were explained by the fact that maspin could contribute to bladder cancer development through DNA methylation and histone deacetylation [83].

Another recent study, focused on the role of Maspin in squamous cell carcinoma (SCC) and transitional cell carcinoma (TCC) of urinary bladder.

In 134 bladder cancer patients, the relationship between clinico-pathological features and Maspin was examined and a high Maspin expression was found in low grade and advanced stage. These results indicated that Maspin expression might predict a better prognosis for bladder carcinoma and it could play a role in tumor progression [84].

Blandamura et al. evaluated, instead, maspin expression in 111 bladder urothelial papillary neoplasms. Their results showed that a strong expression of maspin is related to a better outcome of papillary neoplasms and this is apparently in contrast with the absence or reduced maspin staining observed in the tumors of a lower histological grade and at pTa stage. This was probably due to the fact that the lower grade papillary neoplasms do not induce maspin activation, whereas high-grade neoplasms exert maspin action in an attempt to restrict tumor aggressiveness. (Table 3) [85].

**Table 3:** Maspin expression in bladder cancer

**Authors**	**Years**	**N. patients**	**Maspin expression**	**Clinical features/prognosis**
Acikalin et al.	2012	68 (T1 bladder cancer)	Not specified	Positive maspin = longer PFS and less recurrence
				Negative maspin = shorter PFS and more recurrence
Kramer at al.	2010	162 (pTa-pT1 bladder cancer)	75.9% positive maspin	Positive maspin = PFS: 46 months
			24.1% negative maspin	Negative maspin = PFS: 18 months
Friedrich et al.	2004	110 (pTa-pT1 bladder cancer)	33.6% positive maspin	Positive maspin = PFS: 29 months, MVD: 17.7(CD34) and 6.0 (CD105)per field
			66.4%negative maspin	Negative maspin = PFS: 23 months, MVD = 21.7 (CD34) and 4.2 (CD105)per field
Sugimoto et al.	2004	65 (22 transurethral resection and 43 radical resection specimens)	18.2%positive maspin in transurethral resection specimens	Positive maspin = progression from invasive bladder cancer
			51.2% positive maspin in radical resection specimens	
Nehad et al.	2010	134 (56 squamous cell carcinoma (scc)and 78 transitional urinary bladder(tcc))	53.7% positive maspin (42.8% scc and 61.5% tcc)	Positive maspin = 91.7% low grade
			46.3% negative maspin	Negative maspin = 54.8% high grade
Blandamura et al.	2008	66 (48 pTa e 18 pT1)	38% positive maspin	60.5% positive maspin have high grade
			62% negative maspin	22.44% positive maspin have low grade

In conclusion, further studies are needed to define the role of maspin in clinical practice of bladder tumors.

### Lung cancer

Several studies demonstrated that maspin inhibits the survival pathway by influencing the response to cell death in lung cancer cells [86].

Bircan et al. investigated maspin in 63 patients with different histological lung carcinoma. The mean percentage of maspin expression was significantly higher in squamous cell carcinoma and adenocarcinoma, than in small cell lung cancer (SCLC) [87].

Recently, in lung cancer, maspin biological functions have been linked to its subcellular localization. Specifically, a nuclear, opposed to a combined nuclear and cytoplasmic localization has been associated with increased survival in non-small cell lung cancer (NSCLC). Lonardo et al., Frey et al., Woenckhaus et al. and Hirai et al., examined 123 NSCLC, 80 adenocarcinoma, 487 tissue microarrays and 112 specimens, respectively. They found that squamous cell carcinoma showed the highest expression and almost exclusively a combined nuclear-cytosolic stain in early stage. In contrast, nuclear maspin alone, correlated with favorable clinico-pathologic features and improved prognosis especially in adenocarcinoma [88-90].

Recently our group showed that maspin expression, with nuclear or cytoplasmic localization, together with smoking history, represented prognostic factors in NSCLC. In particular a significant longer overall survival (OS) was seen in patients with higher compared with lower expression of nuclear maspin, and poorer OS was present in patients with a higher intensity of cytoplasmic staining [91].

In another recent study, Wu et al. analysed the expression of maspin in NSCLC and its relationship to vasculogenic mimicry (VM). A total of 160 specimens of NSCLC were considered in this study. The loss of maspin expression may contribute to the invasion and metastasis of NSCLC and it has a positive relationship to VM in NSCLC [92].

Again, Nakagawa et al., Katakuta et al. and Takanami et al. investigated 210 consecutive patients with stage I-IIIA NSCLC, 55 resected NSCLC patients and 181 patients with curatively resected NSCLC respectively. Enhanced maspin expression was found a significant and independent factor in predicting a favorable prognosis in lung squamous cell carcinoma (Table 4) [93-95].

**Table 4:** Maspin expression in lung cancer

**Authors**	**Years**	**N. patients**	**Maspin expression**	**Clinical features/prognosis/predictive factors**
Bircan et al.	2010	28 (squamous cell lung cancer)	89.3% positive maspin	
		18 (lung adenocarcinoma)	77.8% positive maspin	
		17 (small cell lung cancer)	52.9% positive maspin	
Lonardo et al.	2005	46 (squamous cell lung cancer)	100% positive maspin	Positive maspin = almost exclusively nuclear position
		77 (lung adenocarcinoma)	93,5% positive maspin	Positive nuclear maspin in squamous cell lung cancer = low grade, low proliferative rate, absence of invasion, negative p53 vs nuclear-cytoplasmic position.
Frey et al. 2009	2009	80 (lung adenocarcinoma)	93.7% positive maspin	
			62.6% positive nucleus	Positive nuclear maspin = 36.6% proliferative rate, Stage I OS: 87.7 ± 6.9 months, 42.5% moderate and poor differantation, 25,5% p53+, 4.2% high VEGF
			37.3% positive cytoplasm - nucleus	Positive cytoplasmic maspin = 56.9%proliferative rate, Stage I OS: 58.7 ± 6.5 months, 71.4% moderate and poor differantation,53.5% p53+, 39.2% high VEGF
Woenckhaus et al.	2007	487 (tissue microarrays)	72.3% positive maspin	
			65.3% positive nucleus	Positive nuclear maspin = 63.9% squamous cancer, 16.9%
			37.8% positive cytoplasm	Positive cytoplasmic maspin = 78.2% squamous cancer, 6.8% adenocarcinoma adenocarcinoma
Hirai et al.	2005	112 (non-small cell lung cancer)	55.3%positive maspin	Positive maspin = 77.8% positive cytoplasm Stage III and 36.2% positive cytoplasm Stage I
			44.7% negative maspin	
Berardi et al.	2010	439 (non-small cell lung cancer)	85.6% positive maspin	Positive maspin = longer OS
			22.8% positive nucleus	Positive nuclear maspin = independent prognostic factor
			44% positive cytoplasm	Positive cytoplasmic maspin = especially smokers, lower OS than nuclear position
			14.4% negative maspin	Negative maspin = Lower OS
Wu et al.	2012	160 (non-small cell lung cancer)	48.1% positive maspin	Positive maspin = lower vasculogenic mimicry and microvessel density, longer OS, low Stage, low grade, low lymphnode metastasis
			51.9% negative maspin	Negative maspin = higher vasculogenic mimicry and microvessel density, lower OS, high Stage, high grade, lymphnode metastasis
		20 (normal tissue)	100% positive maspin	
Nakagawa et al.	2006	210(non-small cell lung cancer)	73.7% positive maspin in squamous cancer	Positive maspin = 70.1% 5-years OS
			26.3%negative maspin in squamous cancer	Negative maspin = 41.5% 5-years OS
Katakura et al.	2006	55 (non-small cell lung cancer)	Not found	Positive maspin = 67.7% 5-years OS
				Negative maspin = 41.4% 5-years OS
Takanami et al.	2008	181 (non-small cell lung cancer)	40.8%positive maspin	
			65.7% positive maspin in squamous cancer	Positive maspin in squamous cell lung cancer = 52.2% 5-years OS
			34.3%negative maspin in squamous cancer	Negative maspin in squamous cell lung cancer = 24% OS 5 years OS
			22.8% positive maspin in adenocarcinoma	
			59.2% negative maspin	

### Ovarian cancer

An evidence that maspin can inhibit ovarian cancer invasion has been shown in vitro, nevertheless the role of maspin in ovarian cancer remains to be demonstrated. However it seems that maspin expression level is low on normal ovarian surface epithelium, while ovarian cancer cell lines expressed high to low level of maspin expression that is also correlated with shorter survival in patients with epithelial ovarian cancer. Maspin expression was predominantly located in the cytoplasm and occasionally in the nucleus of epithelium and cancer cells [96,97].

Klasa-Mazurkiewicz at al. examined 168 ovarian tissue and found maspin level significantly higher in patients with borderline tumors and early stages ovarian cancers when compared with healthy tissues those with benign and metastatic tumors. Over-expression of maspin was found to correlate with early stage of disease in non-serous subtypes of ovarian cancer and with a positive response to chemotherapy. A statistically significant longer PFS was observed in women with high as compared with low expression of maspin. Again Moshira et al. examining 68 ovarian specimens: 7 normal tissue, 18 benign, 14 borderline, 46 malignant epithelial ovarian neoplasm, detected the same results about progression disease [98].

As well as in other tumor types, also in ovarian cancer it is important its localization inside the cell in order to define the role of maspin.

Sopel et al. and Solomon et al. studied 132 and 11 invasive epithelial ovarian carcinomas, respectively. Cytoplasmatic maspin expression was correlated with worse clinico-pathologic features and prognosis rather than rare nuclear maspin expression [99,100].

Few studies compared maspin with VEGF in ovarian carcinoma. In a recent study, over-expression of maspin, VEGFC, and VEGFD was significantly associated with unfavorable clinico-pathologic features and poor prognosis in 60 ovarian carcinoma tissues. Similar results were obtained by Sood et al. that assessed maspin expression in 104 ovarian tissue specimens [101].

Bauerschlag et al. investigated the prognostic role of maspin expression in 87 ovarian cancer specimens. There was significant correlation between cytoplasmic maspin expression and OS. Docetaxel- and paclitaxel-resistant ovarian cell lines showed an even higher level of maspin expression, suggesting an unfavorable role of cytoplasmic maspin in ovarian cancer (Table 5) [102].

**Table 5:** maspin expression in ovarian cancer

**Authors**	**Years**	**N. patients**	**Maspin expression**	**Clinical features/prognosis**
Klasa-Mazurkiewicz et al.	2009	76 (ovarian cancer)	82.9% positive maspin	Positive maspin = OS and PFS longer than maspin negative tumor.
		8(Krukemberg tumor)	87.2% positive maspin	
		10 (borderline)	90% borderline	
		42(benign tumor)	78.6% positive maspin	
		32(normal tissue)	53.1% positive maspin	
Moshira et al.	2005	46(ovarian cancer) 14(borderline)	65.9% positive maspin = 69% positive cytoplasm, 3.4% positive nucleus and 27.6% positive nucleus and cytoplasm	
		18(benign tumor)	57.1% positive maspin = 37.5% positive cytoplasm and 62.5% positive nucleus and cytoplasm	
			100% positive cytoplasm	
Sopel et al.	2010	132 (ovarian cancer)	88.6% positive maspin	
			22% positive nucleus	Positive nuclear maspin = low tumor grade, less distant metastasis, low Figo stage, longer OS
			12.9% positive cytoplasm	Positive cytoplasmic maspin = high tumor grade, probably distant metastasis, high Figo stage, shorter OS
Solomon et al.	2006	118 (serous ovarian cancer)	81.4% positive maspin	
			21.2% positive nucleus	Positive nuclear maspin = lower VEGF and COX-2, OS:1803 days
			60.2% positive cytoplasm	Positive cytoplasmic maspin = higher VEGF and COX-2, OS:637 days
			18.6% negative maspin	Negative maspin = OS:1146 days
Bolat et al.	2008	60 (ovarian cancer)	88.3% positive cytoplasm	Positive maspin = high VEGF, high grade, high clinical stage, ascite, lymphnode methastasis
			11.7% positive nucleus–cytoplasm	
Sood et al.	2002	80(ovarian cancer)	71% positive maspin	Positive maspin = shorter OS, 63.3% high grade and 90% ascite
		10 (borderline)	26.6% positive nucleus	Positive nuclear maspin = longer OS
		14 (benign tumor)		
Bauerschlag et al.	2010	87 (ovarian cancer)	Not found	Positive maspin = OS: 28 months and platinum-therapy resistance
				Negative maspin = OS: 57 months

### Colorectal cancer

Maspin has been investigated for its hypothetic implication in the cancerogenesis of colorectal cancer, for its probable association with conventional histo-pathological features and for its potential as an independent predictor of survival and response to adjuvant chemotherapy.

Cao et al. investigated the relationship between chronic inflammatory states and neoplasia in 125 specimens included inflammatory bowel disease (IBD) with different grade of dysplasia and also with invasive colorectal cancer. Maspin was found paradoxically over-expressed in both active IBD and colitis-associated dysplasia compared to either inactive IBD or normal colonic mucosa, suggesting a potential role in disease “flare” as well as neoplastic progression [103].

Other recent studies demonstrated a sequential decreased expression rate from adenoma to metastatic colorectal carcinomas and an inverse correlation with p53 and microvessel density [104-106].

Focusing on the clinico-pathologic features associated with maspin, Umekita et al. studied expression of maspin in colorectal adenocarcinomas from 104 patients and observed that maspin was significantly correlated with the depth of invasion, higher Dukes’ classification and high-grade tumor budding. These results suggest that the expression of maspin may correlate with the aggressiveness of colorectal adenocarcinomas [107].

On the other hand, Fung et al. examined 450 resected colorectal cancer finding a stronger expression in right than in left-sided tumors and a stronger expression in high-grade tumors [108].

Regarding relationship between maspin, other biomarker and a possible target therapy, Gurzu at al. evaluated maspin, p53 and other biomarker expression in 110 cases with colorectal cancer with the aim to correlate maspin with angiogenesis and 5-Fluorouracil (5-FU) therapy. They found a correlation with tumor stage and microsatellite status. Therefore maspin nuclear expression, associated with p53 ones, might be used either to select the high-risk microsatellite stable (MSS) colorectal carcinomas diagnosed in Stage II or those MSI cases which can respond to 5-FU [109].

In another recent study on 156 colorectal cases, significant correlations between cytoplasmic expression and high tumor grade and between nuclear expression, high tumor budding and worse OS, were shown. These findings suggest a compartment-dependent function of maspin in colorectal cancer, which can be useful in identifying stage II cases with a higher risk for fatal outcome with a possible benefit from adjuvant chemotherapy [110].

Again Dietmaier et al. investigated nuclear maspin expression in 172 primary stage III colon cancers showing a significant treatment benefit from 5-FU-based chemotherapy in patients with primary tumors expressing Maspin in the nucleus. These data could be useful, if confirmed in a prospective study, to select patients to receive 5-FU treatment or an alternative (non-5-FU based) adjuvant therapy regime (Table 6) [111].

**Table 6:** Maspin expression in colorectal cancer

**Authors**	**Years**	**N. patients**	**Maspin expression**	**Clinical features/prognosis/predictive factors**
Cao et al.	2005	25 (colorectal cancer and IBD)	88%positive maspin	
		51 (active chronic IBD)	92% positive maspin	
		30 (inactive chronic IBD)	43% positive maspin	
		9(normal mucosa)	11% positive maspin	
Song et al.	2002	66 (colorectal cancer)	75.5% positive maspin	Positive maspin in colorectal cancer = 44.7% mutant p53 expression, microvessel density = 181.1+/−54.2
		24(adenoma)	24.5% negative maspin	Negative maspin in colorectal cancer = microvessel density = 256.1 +/75.4
			91.7% positive maspin	
				Positive maspin in adenoma = 0% mutant p53 expression
Jiang-tao et al.	2009	50 (colorectal cancer)	62% positive maspin	Positive maspin in colorectal cancer = no association with positive lymphnode, higher Duke’s stage or mutant p53 expression
		20 (adenoma)	90% positive maspin	
		20 (normal mucosa)	95%positive maspin	
Zheng et al.	2007	119 (colorectal cancer)	95% positive maspin	Positive maspin in colorectal cancer = no liver metastasis = 89% positive maspin
		22 (adenoma)	93%positive maspin	
		118 (normal mucosa)	69%positive maspin	
Umekita et al.	2006	104 (colorectal cancer)	66%positive maspin (15% = T1-T2 and 78.5% = T3-T4)	Positive maspin = 44.2% absent tumor budding, 32.7% Duke’s stage B
Fung et al.	2010	450 (colorectal cancer)	81%positive cytoplasm 80% positive nucleus	Positive maspin = right colon and high-grade tumor
Gurzu et al.	2012	101 (colorecatal cancer)	60% positive maspin	Positive maspin = Stage II-III
Markl et al.	2010	156 (colorectal cancer stage I-II)	48% positive nucleus	Positive nuclear maspin = pT3 OS = 40 months
			72% positive cytoplasm	Positive cytoplasmic maspin = pT3 OS = 63 months
Dietmaier et al.	2006	172 (colorectal cancer)	44.4% negative nucleus	Negative nuclear maspin = OS: 79.2%, OS after 5FU: 32.5%
			55.6% positive nucleus	Positive nuclear maspin = OS:66.6%, OS after 5FU: 71.7%
			24.1% negative cytoplasm	
			75.9% positive cytoplasm	

### Gastric cancer

In human gastric cancer the molecular aspect of carcinogenesis and progression remains elusive. Data are very few but it seems that maspin up-regulation may cause a retarding cell proliferation [112].

Wang et al. investigated maspin expression in 113 gastric cancer and compared it with clinical parameters, MVD and Caspase-3 expression. Cancer less frequently expressed maspin than normal mucosa and dysplasia. Maspin expression showed a significantly negative correlation with invasive depth, metastasis, Lauren’s and Nakamura’s classification and MVD, but a positive correlation with expression of Caspase-3 in gastric cancer [113].

In another recent study in 152 cases of gastric cancer, an inverse relationship between maspin and p53 expression was documented. Moreover maspin expression showed a negative association with histologic grade, depth of invasion, metastasis, and TNM stage. Interestingly, patients with nuclear and cytoplasmic maspin expression presented a longer survival than those with only cytoplasmic expression (Table 7) [114].

**Table 7:** maspin expression in gastric, pancreatic and gallbladder cancer

**Authors**	**Years**	**N. patients**	**Maspin expression**	**Clinical features/prognosis/predictive factors**
Wang et al.	2004	113 (gastric cancer)	50.5% positive maspin	Positive maspin = T4: 35.9%, metastasis: 34.3%, subtype diffuse: 42.1%, undifferentiated: 40%
			49.5% negative maspin	Negative maspin = T4: 64.1%, metastasis: 65.7%, subtype diffuse: 57.9%, undifferentiated: 60%
Lee et al.	2008	152 (gastric cancer)	71.7% positive maspin	
			29.3% negative maspin	Negative maspin = undifferentiated, high stage, metastasis and invasion depth, positive p53
Liu et al.	2012	102 (pancreatic cancer)	98% positive maspin	
Maas et al.	2001	24 (pancreatic cancer)	96% positive maspin	
		5 (PanIn3)		
			100% positive maspin	
Maesawa	2006	69 (cholelithiasis)	14% positive maspin	
		14 (cholelitiasis + intestinal metaplasia)		
			64%positive maspin	
Kim et al.	2012	101 (gallbladder cancer)	59.4% positive maspin	
		25(adenoma)	100% negative maspin	
		10 (normal gallbladder)		
			100%negative maspin	

On the basis of all the above mentioned results, Maspin expression could be considered as an effective and objective marker to reveal biological behaviors of gastric cancer and it could become an useful marker in the future.

### Pancreatic cancer

Unlike other malignant tumors, precancerous pancreatic lesions and pancreatic cancer present up-regulation of maspin gene expression, therefore maspin could be considered a new factor associated with pancreatic cancer. Liu et al. and Maass at al. examined 102 and 29 specimens of pancreatic tissue and demonstrated that more than 90% of cases of ductal adenocarcinoma as well as all high-grade precancerous lesions (PanIN3) were positive for maspin, and normal pancreatic ducts and low-grade precancerous lesions were usually negative for maspin (Table 7) [115].

These data show that maspin may play an important role in the carcinogenesis, tumor invasion, metastasis, and angiogenesis of pancreatic cancer. Its relationship to carcinoma of the pancreas opens a new angle to the discussion on its function in cancer [116].

### Gallbladder cancer

There are few published data focusing on maspin expression in gallbladder cancer, but there is an evidence of maspin implication in cholelithiasis-intestinal metaplasia-dysplasia-carcinoma sequence and in the early step of gallbladder cancerogenesis.

In a recent study on 69 patients with cholelithiasis and 30 patients with gallbladder cancer without cholelithiasis, a positive immunoreactivity for maspin was significantly associated with the presence of intestinal metaplasia in patients with cholelithiasis. These data could support the assumption that intestinal metaplasia of the gallbladder may predispose to gallbladder carcinoma [117].

Furthermore Kim et al. compared the pattern of maspin expression in 101 early and advanced gallbladder cancers. The positivity of maspin expression was found almost in half of gallbladder cancers, whereas no maspin was expressed in adenomas and normal mucosa of gallbladder (Table 7) [118].

### Head and neck cancer

Maspin seems to be an important prognostic factor also in head and neck cancer. In particular some studies revealed that the subcellular location seems to make the difference. Nuclear expression is a positive prognostic factor rather than cytoplasmic location [119,120].

Yoshizawa et al. and Iezzi et al. investigated the possible correlation between clinico-pathologic findings and maspin expression in 54 and 89 oral squamous cell carcinoma, respectively. Maspin was associated with a better survival rate, but a negative correlation with type of invasion, T-stage, lymph-node metastasis and differentiation grade was found [121-123].

Furthermore the cytoplasmic localization was significantly associated with a high risk of disease disseminating to neck lymph-nodes in 56 consecutive cases of oral carcinoma and the nuclear expression was correlated with lower loco-regional recurrence rate and a longer disease-free survival after treatment in elderly patient with laryngeal carcinoma (Table 8) [124,125].

**Table 8:** maspin expression in head and neck cancers

**Authors**	**Year**	**N. patients**	**Maspin expression**	**Clinical pathological features/prognosis**
Yoshizawa et al.	2011	54 (OSCC)	64.8% positive maspin	Positive maspin = 77.8% OS
			35.2% negative maspin	Negative maspin = 29.4% OS
Yoshizawa et al.	2009	71 (OSCC)	64.8% positive maspin	Positive maspin = 100% low grade, 93.3% no lymphnode metastasis, better OS
Iezzi et al.	2007	89 (OSCC)	Not found	Positive maspin = low grade, negative lymphnode, high stage(?)
Marioni et al.	2008	56 (OSCC)	58.9% positive maspin	
			5.3 % positive nucleus	
			1.8% positive cytoplasm-nucleus	
			51.8% positive cytoplasm	Positive cytoplasmic maspin = 61% pN0 and 33% pN+
			41.1% negative maspin	
Marioni et al.	2011	68 (laryngeal cancer)	Not found	Positive nucleus = 22.7% lower recurrence and PFS: 44.5 ± 27.5 months
				Positive cytoplasm and cytoplasm-nucleus = 44.6% longer recurrence and PFS: 34 ± 27.5 months

In conclusion, maspin may represent an useful marker to identify the potential for progression of head and neck cancer, since lower immunoreactivity is associated with larger tumors and a greater invasive potential. Furthermore in head and neck cancer it is necessary to clarify the mechanisms between maspin expression and progression of tumor in order to apply to clinical applications.

### Thyroid cancer

Maspin expression was investigated also in thyroid neoplasms originating in follicular cells. Some studies showed that neither normal follicular cells nor stromal cells expressed maspin. Papillary and follicular carcinomas expressed maspin in significantly higher incidence than those with pure papillary or follicular patterns. These findings indicate that maspin expression is directly associated with prognosis in this setting [126].

Boltze et al. and Tahany et al. detected maspin in 230 and 63 different histological thyroid tissue, respectively, showing that its expression was typical of papillary thyroid tumor, it was associated with tumor multicentricity, vascular and lymphatic invasion, as well as lymph-node metastasis and it was weakly expressed in follicular cancer and absent in normal tissue and in undifferentiated cancers. Maspin expression is a special feature of papillary thyroid carcinomas; promotor methylation-caused maspin repression plays a major role in gene balance and in the process of tumor determination. Therefore maspin could possibly act as a clinically relevant inhibitor of tumor progression, preventing local invasiveness and further systemic progression of papillary thyroid carcinomas [127,128].

Data regarding the relation between maspin expression and clinico-pathological parameters need to be further confirmed on a larger scale with a longer follow up of patients in order to evaluate its relationship with clinical outcome including OS, disease free survival as well as tumor recurrence (Table 9) [129].

**Table 9:** Thyroid cancer and melanoma

**Authors**	**Years**	**N. patients**	**Maspin expression**	**Clinical features/prognosis/predictive factors**
Boltze et al.	2004	68 (papillary carcinomas)	70.5% positive maspin	Positive maspin in papillary cancer = 2% p53+, 83% 110 months OS, Recurrence free disease: 60 months
		38 (follicular carcinomas)	100% negative maspin	Negative maspin in follicular carcinomas = 80%p53+, 40% 110 months OS, Recurrence free disease :40months
Tahany et al.	2006	63(thyroid specimes)=	28.5%positive maspin	Positive maspin = 72% papillary thyroid, 61,1% positive cytoplasm and positive nucleus, 11.1 % positive nucleus and 27.8% positive cytoplasm,
		25 papillary carcinoma	71.5% negative maspin	
Wada et al.	2004	45 (malignant melanoma)	12.5% positive maspin	
			87.5% negative maspin	Negative maspin in melanoma = 83% trunk, 89% extremites, 89% acral, 86% lentigo maligna melanoma, 100% nodular melanoma, 75% superficial spreading, 100% thickness 1.0-4.0 and >4 mm, 100% II-III-IV stage
Chua et al.	2009	77(malignant melanoma)	59.7% positive maspin	Positive maspin = less microvessel density, 78% thin melanoma, 46% thick melanoma
			35.1% negative maspin	Negative maspin = high microvessel density, 22% thin melanomas, 54% thick melanoma

### Melanoma

In melanoma, Maspin was found to have a tumor-suppressor function. Previous reports showed that early hypermethylation of the Maspin promoter might play a role in silencing Maspin. In particular Maspin promoter activity was significantly increased after the thrombin receptor protease activated receptor-1 (PAR-1) silencing, suggesting that PAR1 negatively regulates Maspin at the transcriptional level Maspin tumor-suppressor gene in the acquisition of the metastatic melanoma phenotype [130-133].

Wada et al. investigated maspin expression in five melanoma cell lines, in a normal human epidermal melanocyte cell line, and in 80 surgically resected tumors. Their results suggest that maspin expression in normal skin melanocytes and melanocytic nevi may be repressed, whereas maspin is aberrantly expressed in a subset of melanoma cells by epigenetic modification. In particular Chua et al. in 77 melanoma samples found maspin presence in the radial growth phase in melanoma, and a lost maspin expression in the transition from the radial growth phase to the vertical growth phase of melanoma (Table 9) [134,135].

### Future perspectives

The expression of maspin might be useful as a prognostic and possibly predictive factor for patients with particular types of cancer and data can guide physicians in selecting therapy. Its expression in circulating tumor cells especially in breast cancer, could be also useful in clinical practice along with other factors, such as age, comorbidities, blood examinations in order to select the best therapy to be carried out.

Detecting maspin mRNA amplification by RT-PCR, several authors, as mentioned above, showed maspin in normal breast epithelial cells and in primary and metastatic breast cells, but not in the peripheral blood of healthy donors. Furthermore, they found the presence of circulating maspin-positive cells, potentially neoplastic, in the peripheral blood of patients with breast cancer undergoing conventional-dose chemotherapy. On the basis of these observations, they assessed a possible mobilizing effect of chemotherapy, delivered at standard doses, on mammary cells of potential neoplastic origin. Thus suggesting that the detection of circulating breast cells could have prognostic significance, as it was associated with a higher risk of early relapse or disease progression for patients with limited or stage IV disease, respectively. The results of these studies should be interpreted with caution, and larger studies with longer follow-up are required to definitely establish the clinical usefulness of the test [136-139].

Moreover, maspin expression was found directly correlated with treatment including carboplatin plus vinorelbine combined with radiotherapy in primary head and neck squamous cell carcinoma [140].

In some cases, the subcellular localization could predict response to chemotherapy. In fact, nuclear maspin expression in patients with stage III colon cancer is associated with response to adjuvant 5-FU-based chemotherapy and could help to select patients whose tumors do not express this molecule that may be candidates for an alternative (non-5-FU-based) adjuvant therapy regimen.

Focusing on the malignancies in which maspin showed a positive prognostic value, therapeutic approaches studied so far aimed to re-activate a dormant tumor suppressor gene by designed transcription factors, to hit the system that inhibits the expression of maspin, to identify natural substances that can determine the activation and the expression of maspin or possible “molecules binds” to introduce maspin in cancer cell and gene therapy capable of up-regulating the maspin in an attempt to reduce primarily the risk of metastasis.

Some authors hypothesized that artificial transcription factors (ATFs), composed of modular zinc finger (ZF) domains and designed to recognize specific sequences in the promoter of a tumor suppressor, would result in a re-expression of the endogenous gene silenced by epigenetic mechanisms in aggressive tumor cells (e.g. in metastatic breast cancer and in non-small cell lung cancer) and also in up-regulation of E-cadherin. Reversal and modification of the tumor suppressor epigenetic state can be achieved by blocking DNA and histone methyltransferases as well as histone deacetylases with different doses of ATFs and chromatin remodeling drugs: the methyltransferase inhibitor 5-aza-2’-deoxycytidine and the histone deacetylase inhibitor suberoylanilide hydroxamic acid. It was found that ATFs act in synergy with inhibitors in reactivating endogenous maspin expression. The strongest synergy was observed with the combination treatment ATF-126 + 5-aza-2’-deoxycytidine + suberoylanilide hydroxamic acid. While drugs can be used to achieve the inhibition of these enzymes in an untargeted manner, the precise mechanisms and the genetic cascades whereby these drugs induce biological changes are currently not completely understood and it seems to depend on cell micro-environment. Furthermore, given that tumor suppressors sustain anti-proliferative and anti-metastatic functions, it is essential to target the native tumor suppressor function in order to develop more effective therapies.

Given the clinical relevance of maspin expression in a variety of epithelial tumors (including breast, prostate, lung and colon cancer), these studies describe a novel approach to target multiple human tumors [141-148].

Recently, PAR-1 has been described to regulate the gap junction protein Connexin 43 and the maspin tumor suppressor gene to promote the metastatic melanoma phenotype. Silencing PAR-1 results in decreased activation of p38 MAPK, allowing for increased binding of c-Jun and Ets-1 transcription factors to the maspin promoter. Increased Maspin expression further inhibits cell invasion, through decreased expression and activity of MMP-2, as well as angiogenesis through decreased VEGF expression [149].

An important role of IL-6TS in decreasing adhesion and increasing motility and migration in prostate cancer, along with its effect on the inhibition of maspin was also found. Therefore, specific targeting IL-6TS in prostate cancer patients, might represent an interesting way to refine the currently available experimental anti-IL-6 therapies since sIL-6R and IL-6 are altered in patients with a worse prognosis. This information may be helpful to identify those patients who could benefit from the anti-cytokine therapy [150].

Recent studies have investigated the use of maspin as a therapeutic agent against cancer. In one study, maspin was found to inhibit cancer growth and metastasis in a breast cancer mouse model through a maspin DNA-liposome therapy. Another study showed the ability of maspin to induce apoptosis in tumor-specific endothelial cells. Taken together, these studies demonstrate the potential use of maspin as a viable anticancer therapeutic agent [151]. Gene therapy focusing on the use of adeno-associated virus (AAV, serotype 2) vector encoding maspin in human prostate cancer was also evaluated. Immunofluorescence double staining for maspin protein and apoptosis in LNCaP tumors showed that the percentage of apoptotic cells in AAV-maspin-mediated maspin-expressing cells was significantly high if compared with that in AAV-GFP-mediated GFP-expressing cells. Moreover, significantly fewer CD31-positive microvessels were observed in AAV-maspin-treated tumors compared with the control tumors. These therapeutic responses were highly correlated to persistent maspin expression in tumors, confirmed by Western blot analysis until at least 56 days after treatment. Furthermore AAV-mediated prolonged maspin expression efficiently suppresses human prostate tumor growth in vivo by apoptosis induction and inhibition of angiogenesis [152].

Focusing on natural substances, it has been showed that curcumin, a hydrophobic polyphenol derived from turmeric (the rhizome of the herb Curcuma longa), is implicated in the inhibition of tumorigenesis with a combination of anti-inflammatory, anti-oxidant, immunomodulatory, pro-apoptotic, and anti-angiogenic properties via pleiotropic effects on genes and cell-signaling pathways at multiple levels, including activation of transcription factors, receptors, kinases, cytokines, enzymes and growth factors.

When curcumin is combined with some cytotoxic drugs or certain other diet-derived polyphenols, synergistic effects have been demonstrated [153-155].

Curcumin has recently received a great deal of attention as a chemoprotective agent for the prevention of colon and breast cancer and it may have clinical application in the prevention of prostate cancer. Curcumin down-regulates the androgen receptor gene not only at the protein level but also in its transcriptional activity in androgen-depended prostate cancer. The suppression of androgen receptor transaction may affect the androgen receptor regulated genes such as maspin and curcumin increase the expression of maspin with dose and time dependence.

The mechanism utilized by curcumin to up-regulate maspin expression is unknown at this time. Nevertheless, the up-regulation only occurs in cells that have wild-type p53. This suggests that the activity of curcumin toward maspin expression may be modulated through the p53 pathway. Two other examples of nutritional compounds that have been reported to enhance tumor suppression and inhibit metastasis through up-regulation of maspin are abalone visceral extract and apple peel extract.

As in the case of curcumin, the cells treated with apple peel extract displayed significant increases in the expression of maspin [156-158].

Also resveratrol (trans-3, 49, 5-trihydroxystilbene), a polyphenolic antioxidant found in peanuts, grapes and red wine possesses significant health benefits. This compound has shown beneficial effects in experimental cancer models, where it suppresses the initiation, promotion and progression of tumors Recent studies have implicated activation of the apoptotic pathway as a mechanism accounting for the antitumor benefits of resveratrol. For example, resveratrol inhibits cell proliferation, induces apoptosis of human prostate carcinoma and acute lymphoblastic leukemia cells and increases maspin levels through Akt pathway [159-163].

## Conclusion

It is important to continue to search for the maspin in different tumor types in order to better define its prognostic significance, to refine the definition techniques of cellular compartmentalization and to evaluate the possible therapeutic implications considering peculiarities of expression of maspin in various cancers (Table 10).

**Table 10:** summary of maspin expression

**Cancer**	**Tumor progression normal to cancer**	**Positive cytoplasm or positive nucleus**	**Prognosis**
Breast cancer	Normal tissue: positive maspin	Positive nucleus = better prognosis	Positive maspin = better/worse prognosis depending of localization and of epigenetic modification
	Cancer tissue +/−		
	Cancer metastasis = negative maspin		
Prostate cancer	Normal tissue:	Positive nucleus increased in invasive cancer	Positive maspin = better prognosis
	Negative maspin		
	HGPI: positive maspin		
	Invasive cancer = negative maspin		
Bladder cancer	Normal tissue:	Positive nucleus = better prognosis	Positive maspin = better prognosis
	Positive maspin		
	Cancer tissue:		
	Negative maspin (T2 → T4 positive?)		
Lung cancer	Normal tissue: positive maspin	Positive nucleus = better prognosis vs nuclear and positive cytoplasm	Positive maspin = better prognosis
	Lung cancer = less positive maspin		
Ovarian cancer	Normal tissue: Negative maspin	Positive nucleus (rare) = better prognosis	Positive maspin = better/worse prognosis depending of localization and of epigenetic modification
	Early stage		
	Ovarian cancer = positive maspin		
	Metastic cancer = less positive		
Colorectal cancer	Normal tissue: negative maspin	Positive nucleus = worse prognosis	Positive maspin = better prognosis, positive response to 5FU therapy
	Dysplasia and cancer = positive maspin		
		Positive cytoplasm better prognosis	
Gastric cancer	Normal tissue: positive maspin	Positive nucleus and positive cytoplasm = better prognosis vs only positive cytoplasm	Positive maspin = better prognosis
	Cancer tissue: negative maspin		
Pancreatic cancer	Normal tissue: negative maspin		Positive maspin = worse prognosis
	Pan In1, Pan In2 = negative maspin		
	Pan In3 and cancer invasive tissue = positive maspin		
Gallbladder cancer	Normal tissue: dysplasia = negative maspin intestinal metastasia and cancer = positive maspin		
Head and neck cancer		Positive nucleus = better prognosis	Positive maspin = better prognosis
Thyroid cancer	Normal tissue adenomas and follicular carcinoma = negative maspin/+ papillary carcinoma = positive maspin		Positive maspin = better prognosis
	Anaplastic and poorly differentiated = negative maspin		
Melanoma malignant	Normal tissue: positive maspin	Positive nucleus = better prognosis	Positive maspin = better prognosis
	Melanoma = positive maspin		
	Metastatic melanoma = negative maspin		

The correlation between the expression of maspin and clinical parameters is different depending on the type of tumor. It is likely that the lack of standardization in assessing the positivity of maspin subcellular level and the need for more studies about it can explain and permit the interpretation of differences. It would be more helpful to evaluate the expression of maspin only as a positive nucleus-cytoplasm to ensure greater uniformity of results.

## Competing interest

All the authors have no financial disclosures to declare.

## Authors’ contributions

RB was responsible for the study design. RB, FM, MP, ZB, AS, MDL, MC, SR, SP, MS were responsible for literature review and interpretation. AO, PM, CP, SC were responsible for literature interpretation. RB and SC were responsible for the writing of the report and the corresponding author had the final responsibility to submit for publication. All authors read and approved the final manuscript.
